# Basis-Set Limit CCSD(T) Energies for Large Molecules with Local Natural Orbitals and Reduced-Scaling Basis-Set Corrections

**DOI:** 10.1021/acs.jctc.4c00777

**Published:** 2024-08-29

**Authors:** Dávid Mester, Péter R. Nagy, Mihály Kállay

**Affiliations:** † Department of Physical Chemistry and Materials Science, Faculty of Chemical Technology and Biotechnology, Budapest University of Technology and Economics, Muegyetem rkp. 3, H-1111 Budapest, Hungary; ‡ HUN-REN-BME Quantum Chemistry Research Group, Muegyetem rkp. 3, H-1111 Budapest, Hungary; § MTA-BME Lendület Quantum Chemistry Research Group, Muegyetem rkp. 3, H-1111 Budapest, Hungary

## Abstract

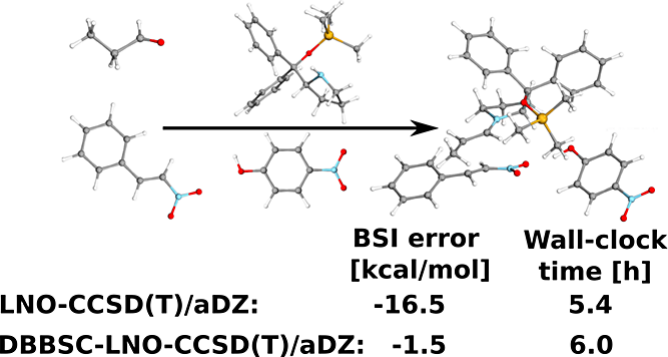

The calculation of density-based basis-set correction (DBBSC), which remedies the basis-set incompleteness (BSI) error of the correlation energy, is combined with local approximations. Aiming at large-scale applications, the procedure is implemented in our efficient local natural orbital-based coupled-cluster singles and doubles with perturbative triples [LNO-CCSD(T)] scheme. To this end, the range-separation function, which characterizes the one-electron BSI in space, is decomposed into the sum of contributions from individual localized molecular orbitals (LMOs). A compact domain is constructed around each LMO, and the corresponding contributions are evaluated only within these restricted domains. Furthermore, for the calculation of the complementary auxiliary basis set (CABS) correction, which significantly improves the Hartree–Fock (HF) energy, the local density fitting approximation is utilized. The errors arising from the local approximations are examined in detail, efficient prescreening techniques are introduced to compress the numerical quadrature used for DBBSC, and conservative default thresholds are selected for the truncation parameters. The efficiency of the DBBSC-LNO-CCSD(T) method is demonstrated through representative examples of up to 1000 atoms. Based on the numerical results, we conclude that the corrections drastically reduce the BSI error using double-ζ basis sets, often to below 1 kcal/mol compared to the reliable LNO-CCSD(T) complete basis set references, while significant improvements are also achieved with triple-ζ basis sets. Considering that the calculation of the DBBSC and CABS corrections only moderately increases the wall-clock time required for the post-HF steps in practical applications, the proposed DBBSC-LNO-CCSD(T) method offers a highly efficient and robust tool for large-scale calculations.

## 1 Introduction

Coupled-cluster (CC) methods^1^ are among the most powerful and accurate tools in quantum chemistry for calculating correlation energies. These approaches systematically account for electron correlation effects through an exponential wave function ansatz, leading to highly precise results. However, a significant drawback of CC methods is their slow convergence with respect to the size of the basis set, which mainly originates from the well-known inability of conventional Gaussian basis sets to account for the electron–electron cusp of wave functions. Achieving high accuracy typically requires the use of very large basis sets, dramatically increasing computational requirements and time.

To address this problem in correlation energy calculations, explicitly correlated methods were developed.^2−4^ By incorporating interelectronic distances into the wave function, these methods significantly improve convergence, achieving chemical accuracy with smaller and more affordable basis sets. The R12 approach,^5,6^ using a linear correlation factor, was initially realized at the second-order Mo̷ller–Plesset (MP2) level.^7^ Later, further progress was made with the introduction of a more sophisticated exponential correlation factor, known as F12,^8,9^ which provides superior results compared to the original formalism. Other developments, including the fixed amplitude,^10^ density fitting (DF),^11^ and complementary auxiliary basis set (CABS)^12,13^ approaches, have led to efficient implementations and successors in the field.^14−16^ As a result, explicitly correlated approaches were also proposed for the highly accurate CC singles and doubles with perturbative triples [CCSD(T)] method.^17−21^

In addition to the above techniques, other methods have been suggested to reduce the computation time associated with the use of extensive basis sets. One such method is the frozen natural orbital (FNO) approximation.^22−24^ In FNO-CC methods, the natural orbitals (NOs) are generated from a lower-level theory, such as MP2,^25^ and those with small occupation numbers are frozen and excluded from the subsequent high-level CC calculations. This significantly reduces the number of active orbitals without substantially sacrificing accuracy, thereby decreasing the computational costs for CCSD(T) calculations. The FNO approximation can also be improved by several correction schemes,^26−28^ and its application has been extended to open-shell systems^28,29^ and higher-order CC methods.^30^ Additionally, a reduced-cost explicitly correlated CCSD(T) approach has also been proposed utilizing FNOs,^31^ further widening the applicability of the method to extended molecular systems.

Local approximations also offer a promising avenue for reducing computational expenses by exploiting the rapid decay of electron–electron interactions with distance.^32−47^ The common feature of these schemes is that the occupied molecular orbitals (MOs) are localized to minimize their spatial extents, and a very compact domain is constructed around each localized MO (LMO), in which most of the important correlation interactions for the given orbital can be described. These domains help eliminate negligible wave function parameters and integrals, thereby accelerating the calculations. The most successful local CC methods also introduce FNO-like approximations and make use of pair- and orbital-specific NO sets to further compress the MO space within the domain.^48−52^ Furthermore, these approaches were combined with F12 techniques to accelerate the basis-set convergence of local CCSD(T) calculations.^53−55^

Another possible way to mitigate the cusp problem is the application of density functional theory (DFT). This approach is well-suited for describing short-range interactions, facilitating the achievement of the complete basis set (CBS) limit with smaller basis sets. The well-established density functional approximations offer an outstanding accuracy-to-cost ratio; however, the biggest drawback of the formalism is that these methods cannot be systematically improved. Consequently, for high-precision applications, the use of DFT is not recommended. Over the past decade, numerous attempts have been made to combine the advantages of DFT and wave function theory (WFT).^56−58^ One of the most promising approaches is the range-separated DFT (RS-DFT) formalism,^59,60^ where the Coulomb operator is divided into long- and short-range components. Since the long-range interactions are effectively handled by WFT, and the semilocal functionals in DFT are good at capturing short-range interactions, this approach successfully diminishes the cusp problem, leveraging the benefits of both methods.^61−70^

In recent years, specifically for improving the description of correlation energy of WFT-based methods, Toulouse, Giner, and their co-workers proposed a density-based basis-set correction (DBBSC) relying on the RS-DFT formalism.^71,72^ The main objective of their correction is to account for the missing part of short-range correlation effects arising due to the incompleteness of the one-electron basis set. To this end, a spatial coordinate-dependent range-separation function was introduced, which effectively quantifies the incompleteness of a given basis set as the function of the spatial coordinate. The final correlation energy correction can be computed in a single and cheap step through this local parameter, greatly improving the correlation energy. The success of the procedure has been demonstrated for thermochemical properties obtained with the CCSD(T) method, and it has also been extended to improve the calculation of other properties, such as dipole moments^73,74^ and excitation energies.^75^

In this paper, we extend the applicability of the DBBSC and CABS corrections to large molecular systems. To this end, we implement the procedure in our local natural orbital (LNO)-based CCSD(T) [LNO-CCSD(T)] scheme,^51,52,76−79^ utilizing local approximations. After a brief overview of the theoretical background, we demonstrate the efficiency of the DBBSC-CCSD(T) method on smaller molecules. Subsequently, for extended systems, we examine the errors arising from local approximations and determine the default values for the truncation parameters. The efficiency of the DBBSC-LNO-CCSD(T) approach is demonstrated through real-life examples of 100–1000 atoms, where the calculated thermochemical properties, reaction energies, and interaction energies are compared with high-quality LNO-CCSD(T)/CBS references.

## 2 Theory

### 2.1 Density-Based Basis Set Correction

The main objective of DBBSC^71,72^ is to approximate the CBS correlation energy of a given method by accounting for the missing part of the short-range correlation effects arising due to the incompleteness of the finite one-electron basis set . For the CCSD(T) approach, the aimed correlation energy can be obtained as
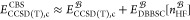
1where *E*_CCSD(T), c_^CBS^ and  are the CCSD(T) correlation energies in the CBS limit and in basis set , respectively. The basis-dependent complementary density functional, , with  as the Hartree–Fock (HF) electron density in , is approximated using a multideterminant (MD) Perdew–Burke–Ernzerhof (PBE) correlation functional^72,80^ as
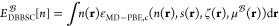
2where *s* is the reduced density gradient, ζ is the spin polarization, and  is the space-dependent local range-separation parameter. The final form of the range-separated correlation functional, ε_MD-PBE,c_ (*n*, *s*, ζ, μ), has been proposed by Giner, Toulouse, and their co-workers^71,72,80,81^ and interpolates between the standard PBE correlation functional,^82^ ε_PBE,c_(*n*, *s*, ζ), at μ = 0 and the exact large-μ behavior^62,83,84^ yielding
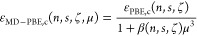
3with
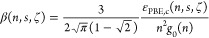
4where *g*_0_(*n*) represents the uniform electron gas on-top pair-distribution function.^84,85^

The dependence of the correction on the basis set arises from the local range-separation parameter, which quantifies the spatial incompleteness of the given basis set . The coupling of DFT and WFT is accomplished by constructing a local real-space representation for the electron–electron Coulomb operator projected onto the chosen basis set.^71^ This general effective two-electron interaction operator, denoted by , can be defined using any arbitrary wave function and pair density. However, as demonstrated in ref (71), the HF wave function suffices to yield reliable results for weakly correlated systems. Consequently, utilizing the frozen core approximation, the space-dependent range-separation parameter is defined as^71,72,81^
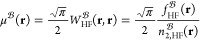
5where
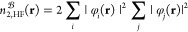
6and
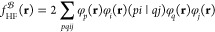
7In the above expressions, *i*, *j*,... refer to correlated (nonfrozen) occupied spin orbitals, *p*, *q*,... are used for generic MO indices including frozen-core orbitals, (*pi*|*qj*) stands for a two-electron integral using the conventional (11|22) notation, while φ_*p*_ (**r**) is the real-space representation of the corresponding MO. To target , the rate-determining step is the construction of the effective operator, particularly, the expression presented in the last equation. Nevertheless, the computational expenses and memory requirements can be decreased by utilizing the DF approximation.^86,87^ In this approach, the matrix **K** with elements *K*_*pi*,*qj*_ = (*pi*|*qj*) is factorized as **K** = **IV**^–1/2^**V**^–1/2^**I**^T^ = **JJ**^T^, with
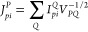
8where *P* and *Q* stand for the elements of the DF auxiliary basis, whereas *I*_*pi*_^*Q*^ and *V*_*PQ*_ are three- and two-center Coulomb integrals, respectively. Using this notation, the final form of the intermediate  can be expressed as
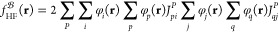
9In practice, eqs 2, 5, 6, and 9 are evaluated using integration grids developed for DFT methods.

The presented approach can efficiently approximate the correlation energy in the CBS limit. However, especially for smaller one-electron basis sets, the HF energy also has a significant basis set incompleteness error. As demonstrated also in the context of DBBSC,^86^ the CABS-corrected HF energies significantly enhance the accuracy of the calculations. Based on this finding, the final energy expression for the DBBSC-CCSD(T) method is obtained as
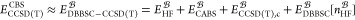
10where  and  are the corresponding HF energy and CABS correction contributions, respectively.

### 2.2 Local Approximations

Molecular orbital localization and local approximations are key techniques for reducing the complexity inherent in correlation energy calculations. By transforming delocalized canonical orbitals obtained from HF calculations to LMOs,^88^ denoted by *I*, *J*,..., the interaction between electrons decreases more rapidly with their distance, and localized orbitals primarily interact with their immediate neighbors.^32,89−91^ This disparity in the significance of electron interactions across a molecule lays the groundwork for our local approximations.^51,52,76−79,92,93^ If one decomposes the correlation energy as the sum of contributions from individual LMOs^35,43,94,95^ and constructs a compact local domain around each LMO that includes all significant interactions, then the number of variables used for the calculations can be drastically reduced, while the error in the final result is negligible.^52,77^

Utilizing effective CCSD(T) implementations based on local approximations, the evaluation of the DBBSC would be the rate-determining step in correlation energy calculations. However, as eq 5 is also invariant to unitary rotations among the occupied orbitals, similar approximations can be used for its evaluation. Assuming that the domains used in our LNO-CCSD(T) scheme^51,77−79^ are sufficiently accurate for this purpose as well, the infrastructure established therein can conveniently be applied in this case. Here, we briefly summarize the steps required to construct such restricted and compact domains, while the detailed descriptions can be found in our previous works.^51,52,76−79^ First, the so-called primary domains (PDs) are constructed around each LMO, hereafter referred to as the central LMO, typically including its immediate environment. To accurately represent the virtual orbitals, projected atomic orbitals (PAOs)^32^ are employed, which are constructed by projecting the atomic orbitals (AOs) onto the space orthogonal to occupied MOs. The PDs are designed to capture the most significant local interactions that directly impact the central LMO, ensuring that primary electron correlation effects are properly represented. After assembling the PDs, pair correlation energies are calculated using pair domains formed as the union of a given LMO pairs’ PDs. The pair correlation energy is evaluated efficiently using multipole expansions,^51,93^ and only the pairs surpassing a specific energy threshold are considered as strong pairs.

Next, the so-called extended domains (EDs), denoted by , are formed, which are pivotal for refining the approximation of electron correlation energies further beyond the scope of pair domains. The ED incorporates the central LMO and all LMOs that form strong pairs with the central LMO. The virtual space of the ED is spanned by the PAOs constructed from AOs that reside on the atoms of the PAO center domain. The latter includes the most important atoms of the ED’s LMOs selected according to the Boughton–Pulay (BP) algorithm.^96^ This ensures that all significant interactions involving the central LMO are captured. To adequately describe the correlation effects beyond immediate strong interactions, the EDs also include additional PAOs that may not directly interact strongly with the central LMO but contribute to the correlation energy through medium to long-range effects, which are crucial for describing the subtleties of the electronic structure. The orthogonality of the selected orbitals within the ED is ensured through Gram–Schmidt–Löwdin orthogonalization technique.^97,98^ Finally, the orbitals of the ED are canonicalized by diagonalizing the Fock matrix in its occupied and virtual space separately. For the evaluation of the Coulomb integrals required for the calculation of the correlation contribution of the central LMO, the DF approximation is employed, but the auxiliary basis contains only the auxiliary functions residing on the atoms of the PAO center domain.

As mentioned, eq 5 can be evaluated using localized orbitals, and the corresponding contributions can be split up as the sum of contributions from individual occupied orbitals:  and . Utilizing the local domain approach described, the corresponding intermediates can be approximated for the *I*th LMO within  as
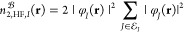
11and
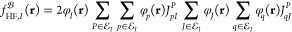
12At this point, an extension of the previous LNO methodology was required, as the summation in the above expression includes core occupied orbitals too, while those are usually left out of the domain construction when the frozen core approach is employed. Here, we first separately localize the core and valence MO subspaces. Then, the core orbitals are selected that are needed in the EDs and the transformation steps yielding the core LMO dependent three-center integrals of the ED. To identify the core LMOs contributing to an ED, we determine their BP domains using a tight truncation criteria also used for the ED atom list construction, that is, 0.9999 by default and governed by the bpedo threshold.^51,77^ As the core LMOs are highly localized, these BP lists are compact (ca. 5–10 atoms). Thus, we can assign a core LMO to an ED if its entire BP list is included in that ED. This strategy may collect some unnecessary core LMOs that are localized closer to the edges of the ED, but their relatively small number brings in negligible additional costs.

We note that, in principle, the above contributions should be evaluated for the entire integration grid. However, efficient prescreening techniques can be introduced. If the value of the central LMO in a grid point, φ_*I*_(**r**), is lower than a predefined threshold, denoted by ε_preI_, the contributions associated with the corresponding grid point can be neglected within the domain. This procedure will be referred to as preI, and it narrows the grid down to the space where the value of the central LMO is non-negligible. In addition, the grid can be further pruned by applying similar prescreenings for φ_*J*_(**r**) when evaluating the summation over *J*, using a different threshold denoted by ε_preJ_, resulting in *J*-selective grid batches for those contributions. This procedure will be referred to as preI+preJ. The efficiency of these schemes will be thoroughly discussed later. Notice that the summations in eqs 11 and 12 are performed over the orbitals and auxiliary functions of the ED. Consequently, thanks to the grid prescreening, their evaluation scales linearly with the system size with either preI or preI+preJ. Of course, the prescreening of the φ_*I*_(**r**) values is quadratically scaling but its time demand is negligible compared to the other steps of the calculation.

According to eq 10, the CABS correction is also required to calculate the DBBSC-CCSD(T) total energy. In this case, the rate-determining step is the evaluation of the Fock matrix within the space spanned by the HF MOs and the CABS virtuals. Since the CABS is fairly large, this step could pose a serious limitation for extended systems. To avoid this problem, the local DF (LDF) approximation is used for the exchange contribution of the Fock matrix construction.^93,99−102^ That is, localized occupied MOs are used at the construction of the exchange matrix, and for each LMO, a fitting domain is assembled that includes only a limited number of DF auxiliary functions. To that end, Löwdin atomic charges are computed for the LMOs, and all atoms with a charge greater than 0.05 are selected. Additionally, all other atoms are included in the fitting domain of the LMO for which the electron repulsion integrals involving the corresponding AOs and the basis functions residing on the atoms selected in the first step are estimated to be greater than a predefined threshold denoted by ε_LDF_. The fitting functions in the restricted local domain are then included according to this atom list, and these functions are applied to approximate the Coulomb integrals involving the occupied LMO. This approximation formally reduces the quartic-scaling scaling of the exchange computation to cubic. The scaling can be reduced to even linear if further domain approximations are employed for the AOs.^93,101,102^ Hereinafter, if the local approximations are utilized, we will refer to the DBBSC-CCSD(T) method as DBBSC-LNO-CCSD(T).

To accelerate basis-set convergence, a rational alternative to the presented scheme could be the F12-based CC methods using local approximations.^53−55^ However, the development and implementation of these excellent approaches are quite complicated and challenging. In contrast, the advantage of the presented approach is its ease of implementation into existing frameworks, and its favorable computational and memory requirements. Our goal with this development is to enable the fast and efficient description of extensive molecular systems where the single-determinant representation is adequate. Otherwise, neither the CCSD(T) method nor the use of the HF wave function in eq 5 can describe the system properly, and more advanced multireference-based approaches are suggested.^103^

## 3 Computational Details

### 3.1 Methods and Basis Sets

All calculations were carried out using the development version of the Mrcc suite of quantum chemical programs.^104,105^ The technical details of our explicitly correlated,^21,31^ local approximation based,^51,52,76−79,92,93^ LDF,^93,102^ and DBBSC^86^ implementations were discussed in our previous studies. The reference explicitly correlated CCSD(T) calculations were performed with the CCSD(F12*) approach of Hättig et al.^20^ in conjunction with our (T+) correction^21^ [CCSD(F12*)(T+)].

In this study, as the AO basis set, the correlation-consistent aug-cc-pV*X*Z (*X* = D, T, Q)^106−110^ and Karlsruhe basis sets, such as def2-SVPD and def2-TZVP(PD),^111,112^ were employed. For the sake of brevity, the aug-cc-pV*X*Z basis sets will be referred to as a*X*Z. For the CABS, the “OPTRI” bases of Yousaf and Peterson^113,114^ were applied. The choice is straightforward for a*X*Z basis sets, while the aDZ-OPTRI and aTZ-OPTRI CABS were used for def2-SVPD and def2-TZVP(PD), respectively. The DF approximation was invoked at both the HF and the post-HF levels. Where the CABS correction or explicit correlation were not applied, the corresponding fitting bases of Weigend^115,116^ were employed, otherwise, the aug-cc-pV(*X* + 1)Z-RI-JK and the aug-cc-pwCV(*X* + 1)Z-RI basis sets^117^ were used, respectively. The frozen core approximation was utilized in all post-HF calculations. For DBBSC, mainly the Treutler–Ahlrichs (TA) numerical quadratures^118^ were employed together with the Log3 radial grid of Mura and Knowles,^119^ while the very fine default adaptive integration grid of the Mrcc package was also used for cross-validation. The reported computation times are wall-clock times determined on a machine with 256 GB of main memory and an AMD EPYC 7763 processor using 8 cores.

### 3.2 Benchmark Sets and Large-Scale Applications

To briefly assess the performance of approaches, the test set of Knizia, Adler, and Werner (KAW)^18^ was used for benchmark calculations. This well-established compilation is often employed to test explicitly correlated methods, covering a wide range of difficult examples, which includes 49 atomization energies and 28 and 48 reaction energies of closed- and open-shell systems, respectively, involving 66 species. The reference CBS values are two-point extrapolated CCSD(T) a(5,6)Z energies taken from previous works.^21,86,120^

As extended molecular systems, well-established representative examples were selected. The default values of the parameters introduced in this study (ε_preI_, ε_preJ_, and ε_LDF_) were determined using a single DNA adenine-thymine base pair (DNA_1_)^121,122^ with the aDZ and aTZ basis sets and the vancomycin molecule^49^ with the def2-TZVP basis set.

The performance of the DBBSC-LNO-CCSD(T) method was tested with respect to the LNO-CCSD(T)/CBS references, with a primary focus on thermochemical and kinetic properties, as well as interaction energies. Accordingly, barrier heights were calculated against an a(Q+d,5+d)Z extrapolated reference for a halocyclization reaction.^123,124^ Here, an intramolecular nucleophilic addition is induced on an olefin by the addition of a halogen dichloro-dimethylhydantoin to the double bond via a base (quinuclidine) catalyst. Additionally, an organocatalytic Michael addition was selected, where the reaction of propanal and β-nitrostyrene is facilitated by a diphenylprolinol silyl ether catalyst and a *p*-nitrophenol cocatalyst. In this case, the largest species along the reaction path was inspected, that is, the transition state of the carbon–carbon bond formation, for which a(T,Q)Z reference is available.^77,125^ Both examples exhibit the difficulties of forming a transition state complex from 3–4 similar sized reactants and catalyst(s) prone to serious basis set superposition error, as well as multiple simultaneous bond formation and breaking steps.

Reaction energies were also assessed. First, isomerization energies were computed for the fourth reaction of the isomerization test set (ISOL4) by Grimme and co-workers using a(Q,5)Z reference.^77,126^ In this challenging case, the two intermediate steps in a biosynthesis are markedly different, so one cannot rely on any error compensation between the species. Second, the AuAmin organometallic reaction^77,127^ was studied using a(Q+d,5+d)Z reference. This reaction poses a significant challenge for local correlation methods because of the extensive contribution of numerous important but individually small noncovalent interactions.

**Figure 1:**
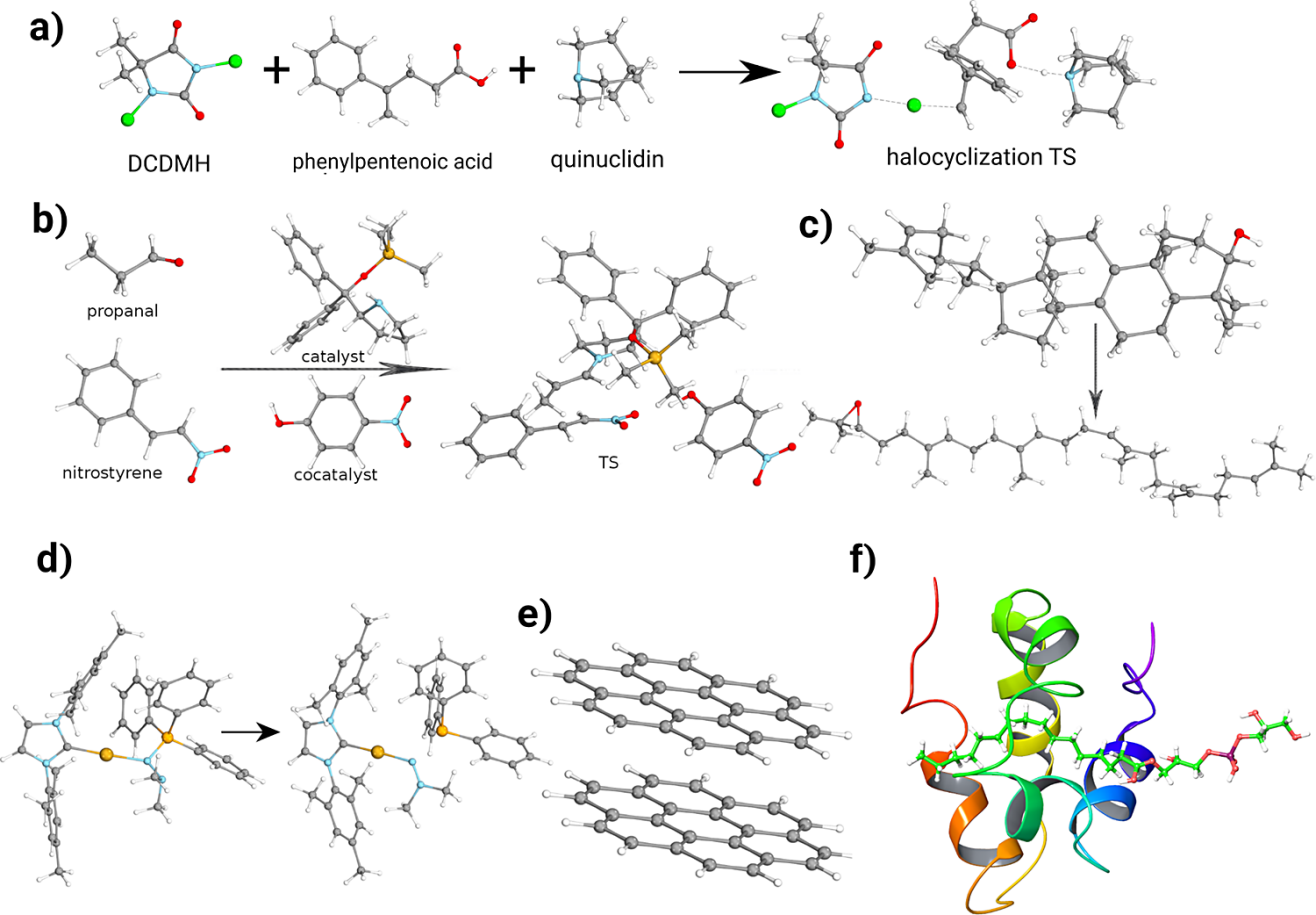
Illustration of (a) halocyclization transition state, (b) Michael addition transition state, (c) ISOL4 isomerization reaction, (d) AuAmin organometallic reaction, (e) coronene dimer, and (f) lipid transfer protein complex.

Additionally, interaction energies were also inspected. For this purpose, the notoriously complicated coronene dimer of the L7 set^128^ was selected, employing a(Q,5)Z values as reference.^77,129^ The dimerization energies of extended molecules with large interacting surfaces, especially for extended and polarizable π–π interactions, are known to exhibit slow basis-set convergence. Furthermore, large-scale calculations illustrating the current capabilities of the DBBSC-LNO-CCSD(T) implementation are presented for a lipid transfer protein (LTP),^130^ containing 1023 atoms, where def2-(T,Q)ZVPPD reference is available. Here, in order to make a comprehensive comparison, both counterpoise (CP)-corrected and CP-uncorrected values will be discussed. In these benchmark calculations, our default settings were applied to the local domain construction.^77^ The systems used to demonstrate the efficiency of the present method are collected in Tables 1 and 2, while their graphical representations is available in Figure 1. The chemical properties discussed above were calculated from the total energies. The Supporting Information includes all raw numerical data.

**Table 1:** CBS Energies (in kcal/mol) Used in the Large-Scale Applications

name	type	CBS energy	CBS reference
halocyclization	barrier height	9.06	a(Q+d,5+d)Z
Michael addition	barrier height	–4.81	a(T,Q)Z
ISOL4	isomerization energy	69.52	a(Q,5)Z
AuAmin	reaction energy	–49.56	a(Q+d,5+d)Z
coronene dimer	interaction energy	–25.60	a(Q,5)Z
LTP	interaction energy	–12.48	def2-(T,Q)ZVPPD

**Table 2:** Sizes of the Largest Species Used in the Large-Scale Applications

name	number of atoms	basis set	total AO function	total CABS functions
halocyclization	63	a(D+d)Z	1033	2892
		a(T+d)Z	2203	3395
Michael addition	90	aDZ	1472	4190
		aTZ	3155	4913
ISOL4	81	aDZ	1163	3239
		aTZ	2576	3868
AuAmin	92	a(D+d)Z	1526	4325
		a(T+d)Z	3248	5085
coronene dimer	72	aDZ	1320	3840
		aTZ	2760	4440
LTP	1023	def2-SVPD	14,730	46,720

## 4 Results and Discussion

### 4.1 Performance of DBBSC-CCSD(T) for Smaller Systems

**Figure 2:**
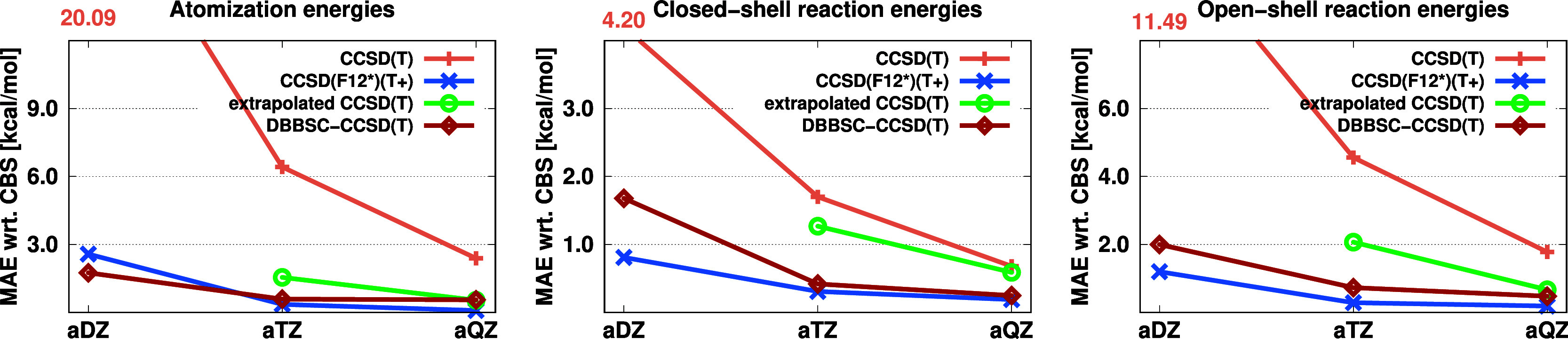
MAEs (in kcal/mol) of the KAW test set^18^ for the standard, F12, a[(*X* – 1), *X*]Z extrapolated, and DBBSC-CCSD(T) methods using various basis sets.

First, we briefly evaluate the performance of DBBSC-CCSD(T) without local approximations. Though DBBSC-CCSD(T) was thoroughly benchmarked in our previous paper,^86^ particular aspects, such as its performance in comparison to basis set extrapolation or its behavior with Karlsruhe-type basis sets, were not considered, and these are important from the point of view of the present study. Here, we discuss the mean absolute errors (MAEs) of atomization and reaction energies of the KAW test suite using various basis sets. To avoid any potential issues arising from the selection of the numerical quadrature, the very fine TA5 grid was used for all calculations discussed in this subsection. First, the performance of DBBSC-CCSD(T) is assessed in comparison with the CCSD(F12*)(T+) method and the a[(*X* – 1), *X*]Z extrapolated CCSD(T) approach. For the latter, the common two-point extrapolation formulas are used.^131,132^ The numerical results are depicted in Figure 2.

We would like to emphasize that the trends observed for various thermochemical properties are fairly consistent, and accordingly, only a short summary is presented. Similar results were discussed in detail in our previous work.^86^ For atomization energies, the DBBSC-CCSD(T) method exhibits surprising accuracy using even the smallest basis set, with a MAE of 1.8 kcal/mol. With increasing cardinal numbers, the performance of DBBSC-CCSD(T) remains competitive, closely approaching the results obtained by the somewhat more demanding CCSD(F12*)(T+) method. Using the aTZ basis set, the MAEs are 0.6 and 0.4 kcal/mol for DBBSC-CCSD(T) and CCSD(F12*)(T+), respectively. The extrapolated CCSD(T) approach also shows significant improvements in comparison with the standard method; however, a(T,Q)Z extrapolation is required for precise results as the error is still higher than 1.6 kcal/mol with a(D,T)Z.

For closed-shell reaction energies, CCSD(F12*)(T+) consistently provides the best results, with the lowest errors across all basis sets. The MAE is already below 1.0 kcal/mol using the aDZ basis set, while it drops to 0.3 and 0.2 kcal/mol with aTZ and aQZ, respectively. The DBBSC-CCSD(T) method shows somewhat larger errors using the double-ζ basis, while the difference in the MAEs becomes very small, less than 0.1 kcal/mol, for the larger basis sets. Interestingly, the performance of the extrapolated CCSD(T) approach is less satisfactory. In this case, the results barely surpass those obtained with the standard CCSD(T) method. With the a(D,T)Z extrapolation, the error is 1.3 kcal/mol, while the error decreases to only 0.6 kcal/mol when a(T,Q)Z is used. The rankings are similar for the open-shell reaction energies. Accordingly, CCSD(F12*)(T+) is the most precise method, where the MAE is 1.2 kcal/mol using the aDZ basis set, while it rapidly drops to 0.3 kcal/mol with aTZ. In this case, the difference between the CCSD(F12*)(T+) and DBBSC-CCSD(T) approaches is somewhat lower using smaller basis sets; however, this difference does not disappear for larger basis sets. Nevertheless, the improvements are significant in comparison with the standard CCSD(T) approach as the MAEs are 2.0, 0.7, and 0.5 kcal/mol with the aDZ, aTZ, and aQZ basis sets, respectively. This finding is also true for the extrapolated CCSD(T) method, but the improvements are somewhat less pronounced. In general, we can conclude that the DBBSC-CCSD(T) approach does not strictly outperform the explicitly correlated CCSD(F12*)(T+) method, although the results are close. This finding is particularly true when we examine the results obtained with the aTZ basis set. Nevertheless, what makes the DBBSC-CCSD(T) method desirable is that the required wall-clock times are 40% lower in comparison with the explicitly correlated approach.^86^ The extrapolated CCSD(T) also shows significant improvement over the standard approach; however, it is not competitive against the former methods.

The performance of the standard and DBBSC-CCSD(T) approaches is well-known using correlation-consistent basis sets. In order to gain a broader understanding of the application of different basis sets, we briefly assess the capabilities of the Karlsruhe basis sets. These results are collected in Table 3. Inspecting the atomization energies, comparing the aDZ and def2-SVPD sets, a noticeable improvement can be observed in the MAE for standard CCSD(T), where the MAE drops from 20.2 kcal/mol with aDZ to 14.9 kcal/mol with def2-SVPD. For DBBSC-CCSD(T), a modest improvement is shown from 1.8 to 1.7 kcal/mol, supporting that DBBSC is already quite efficient, even with smaller basis sets. Moving to aTZ and def2-TZVPPD, the results attained with the correlation consistent basis sets are somewhat better; however, the difference is only 0.1 kcal/mol even for the standard CCSD(T) method. As expected, the def2-TZVP basis set provides higher accuracy in comparison with def2-SVPD, but the additional polarization and diffuse functions are required for precise calculations.

**Table 3:** MAEs (in kcal/mol) of the KAW Test Set^18^ for the Standard and DBBSC-CCSD(T) Methods Using Various Basis Sets

basis set	atomization energies		closed-shell reaction energies		open-shell reaction energies	
	CCSD(T)	DBBSC-CCSD(T)	CCSD(T)	DBBSC-CCSD(T)	CCSD(T)	DBBSC-CCSD(T)
aDZ	20.18	1.75	4.25	1.68	11.54	2.00
aTZ	6.45	0.59	1.71	0.42	4.59	0.73
def2-SVPD	14.90	1.70	5.94	2.08	8.32	2.17
def2-TZVP	10.53	1.38	3.83	1.17	5.70	1.07
def2-TZVPPD	6.49	0.70	1.25	0.60	3.89	0.94

For closed-shell reaction energies, a quite different trend can be observed. For DBBSC-CCSD(T), the aDZ and aTZ basis sets provide slightly more reliable results in comparison with the corresponding def2-SVPD and def2-TZVPPD counterparts, respectively. The differences are not significant, being around 0.3 kcal/mol in both cases. For the standard CCSD(T) method using a double-ζ basis, the correlation consistent basis sets are better, while for triple-ζ ones, def2-TZVPPD is the winner, although the differences are not significant here either. In this case as well, it is true that increasing the size of the basis set monotonically reduces the errors. Similar findings can be observed for open-shell reaction energies. Again, for DBBSC-CCSD(T), somewhat higher accuracy can be achieved using the correlation-consistent basis sets, but the difference does not exceed 0.2 kcal/mol in either case. Conversely, for standard CCSD(T), somewhat better results can be achieved with the def2-SVPD and def2-TZVPPD basis sets. In general, we can conclude that, especially for DBBSC-CCSD(T), significant differences between the basis sets cannot be observed when examining those of the same quality. Additionally, the MAE decreases with increasing size of the basis set, and the def2-TZVP set can be a suitable alternative with an accuracy lying between double- and triple-ζ basis sets supplemented with diffuse functions. The benefit is that the size of def2-TZVP is about two-thirds of that of aug-cc-pVTZ.

### 4.2 Determining Default Truncation Parameters

**Figure 3:**
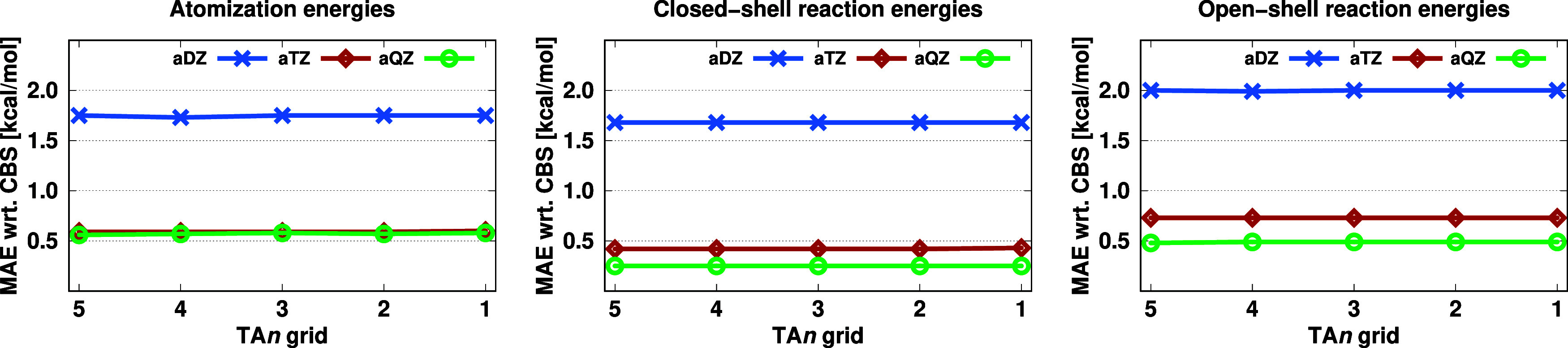
MAEs (in kcal/mol) of the KAW test set for the DBBSC-CCSD(T) method using various basis sets and TA grids.

The calculation of the range-separation function scales as *N*_grid_*N*_occ_^2^*N*_basis_^2^, where *N*_grid_, *N*_occ_, and *N*_basis_ are the number of grid points, number of occupied MOs, and the total number of HF MOs, respectively. Since the number of grid points is very large for common DFT applications, it is worth examining how dense numerical quadrature is necessary to evaluate the current correction in order to minimize computational requirements. Accordingly, we provide a short overview of the grid-requirement of the DBBSC-CCSD(T) method, illustrating how accuracy varies with different TA*n* grids and basis sets for atomization energies, as well as closed-shell and open-shell reaction energies. The numerical results using different quadratures are presented in Figure 3. For atomization energies, the results obtained with all the basis sets exhibit minimal variation across different TA quadratures. Accordingly, the MAEs are fairly unchanged, remaining around 1.75 kcal/mol using the aDZ basis set and 0.60 kcal/mol with both the aTZ and aQZ basis sets, across all grids. The largest deviation in the MAEs is 0.02 kcal/mol, which is highly acceptable. Similarly, for closed-shell reaction energies, the errors are remarkably consistent. With the aDZ basis set, the MAE is close to 1.70 kcal/mol, while it is consistently around 0.40 and 0.25 kcal/mol with aTZ and aQZ, respectively, regardless of the quadrature applied. The errors for open-shell reaction energies follow the same trend, with almost no fluctuation in the MAEs among different TA grids. Again, the MAEs are approximately 2.00 kcal/mol with the aDZ and about 0.75 and 0.50 kcal/mol with the aTZ and aQZ basis sets, respectively, for all quadratures. Inspecting the reaction energies, the largest difference does not exceed 0.01 kcal/mol. Based on these findings, we can conclude that the nearly unchanged MAEs across various TA quadratures in all types of properties imply that the smallest grid, TA1, is sufficient for practical applications within the DBBSC-CCSD(T) scheme. Since more dense grids do not improve accuracy, using TA1 minimizes computational requirements and time without sacrificing precision. These findings will be verified on a larger example in the following.

**Figure 4:**
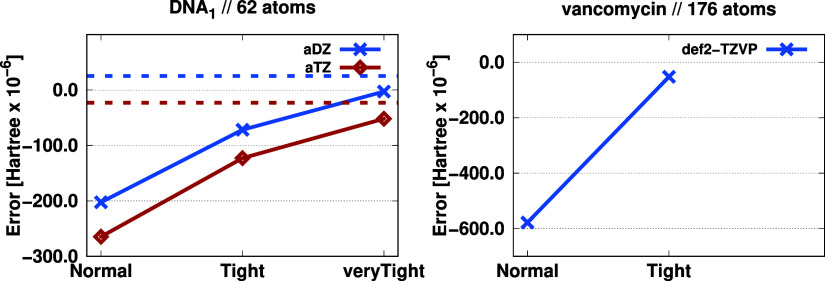
Error (in μE_h_) of the DBBSC (solid) as a function of the domain construction parameters. The dashed lines indicate the error of the TA1 grid compared to our highly accurate adaptive grid. See text for further details.

Next, we determine the magnitude of the errors arising from our local approximations. For this purpose, extensive studies were carried out for the 62-atom DNA_1_ and the 176-atom vancomycin molecules. For the former, reference DBBSC calculations were performed without any approximations using our very fine adaptive grid. First, cross-validation was accomplished by inspecting how the error changes when the TA1 quadrature is used. Then, we examined the errors arising from our local approximation with the continuous tightening of the parameters related to domain construction. These default parameter sets, labeled as Normal, Tight, veryTight..., were determined and adjusted for our high-precision LNO-CCSD(T) calculations.^77^ The calculations were carried out using both the aDZ and the aTZ basis sets. For the vancomycin molecule, the examination of tightening parameter settings was also accomplished; however, due to the size of the molecule, the reference DBBSC was calculated using the TA1 grid with a veryTight set of domain construction parameters. In this case, the def2-TZVP basis set was used. The results are depicted in Figure 4. First, the results obtained for the DNA_1_ molecule are discussed, where the DBBSCs are −1.44003 and −0.58212 E_h_ using the aDZ and aTZ basis sets, respectively. As can be seen, using a smaller quadrature causes negligible error. The difference between the results obtained with TA1 and the very dense adaptive grid is only 25 μE_h_, which is less than 0.5 μE_h_/atom. This finding is true with both basis sets. The advantage of using the smaller TA1 quadrature is evident as in this case, 15- and 20-times fewer grid points are needed with the aDZ and aTZ basis sets, respectively. The error arising from the domain construction is well-balanced with both basis sets. Using the Normal parameter set, the error is approximately 250 μE_h_, which is 4 μE_h_/atom. By tightening the parameters, the error decreases monotonically, reaching only 100 μE_h_ with the Tight criterion, and practically vanishes with the veryTight parameters. When applying the Normal parameters, the relative error in DBBSC is around 0.02% and 0.04% for the aDZ and aTZ bases, respectively.

Since we observed that the error disappears with the veryTight parameters, using this DBBSC, being −1.98980 E_h_, as a reference, appears appropriate for the vancomycin molecule. Here, the error for the default domain construction is around 600 μE_h_, which translates to 3 μE_h_/atom, while the relative error is 0.03% in this case. As can be seen, tightening the parameters leads to rapid error convergence, with the Tight parameter set resulting in an error of 50 μE_h_. Based on these results, we can conclude that the domain construction designed for LNO-CCSD(T) calculations is also suitable for DBBSC. The domain construction with the Normal parameters and the TA1 grid can be reliably used for larger systems with basis sets of various quality.

**Figure 5:**
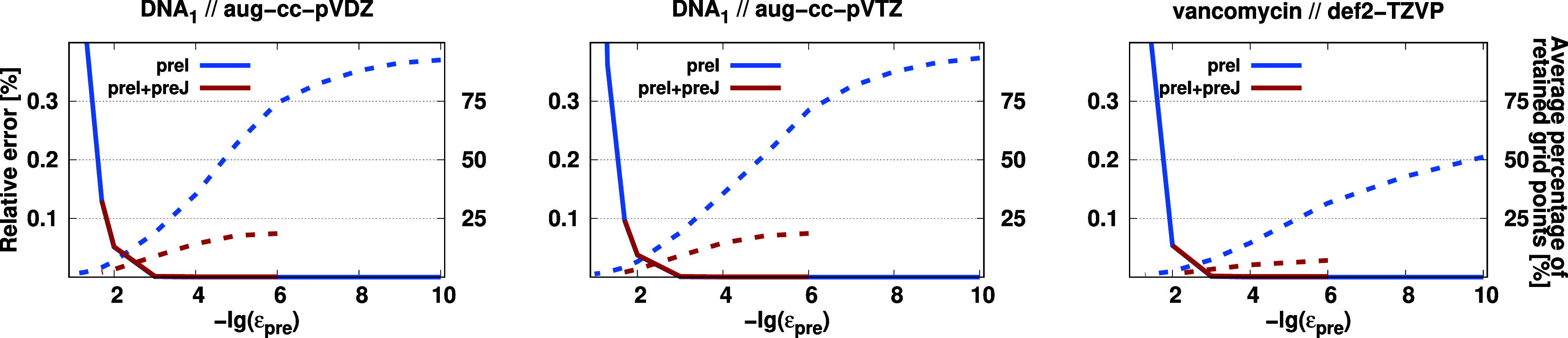
Relative error of the DBBSC (solid) and the average percentage of retained grid points (dashed) for various systems as a function of the corresponding truncation parameters. See text for further details.

Next, the prescreening of the grid will be scrutinized employing the same molecules and the TA1 quadrature. The results are summarized in Figure 5. As can be seen, regardless of the basis set, the same results were obtained for the DNA_1_ molecule. Consequently, these results are discussed together. First, let us consider the preI scheme. Inspecting the errors, it can be stated that the approximation is practically error-free up to a threshold value of ε_preI_ = 10^–3^. At this point, the deviation from the reference is approximately 5–10 μE_h_, which is highly acceptable. The error then begins to increase, but even at ε_preI_ = 10^–2^, the relative error does not exceed 0.05%. Regarding the number of retained grid points, it can be concluded that with increasing threshold value, as expected, it gradually and smoothly decreases. Using ε_preI_ = 10^–3^, only 20% of the total grid is retained, allowing the calculation of contributions to be performed five times faster in this case.

With the preI+preJ scheme, the grid size can be further compressed selectively for each *J*-dependent contribution (see eq 12). In this case, the threshold ε_preI_ was fixed at 10^–3^. Accordingly, only the additional error is calculated. Changing the value of ε_preJ_ yields completely similar results as in the previous case. It can be stated that the approximation is practically error-free up to ε_preJ_ = 10^–3^, with the additional error being approximately 10–20 μE_h_. Using these conservative thresholds, for the calculation of *J*-dependent contributions, which are the rate-determining steps within the domain, only 10% of the total grid is used.

Similar results were obtained for the vancomycin molecule using the def2-TZVP basis set. Again, the preI and preI+preJ schemes are practically error-free up to ε_preI_ and ε_preJ_ = 10^–3^. The error is around 20 μE_h_ for preI, while the additional error amounts to 40 μE_h_ with the preI+preJ prescreening. Considering the magnitude of relative errors, these inaccuracies are negligibly small. Based on these results, we chose a default value of 10^–3^ for the prescreening parameters. Examining the number of the retained grid points for the vancomycin molecule, we can conclude that higher speedups can be gained in this case. This is not surprising as vancomycin is larger, but the extent of the LMOs remains similar. With the preI scheme, 8% of the grid points are retained, while with the second prescreening, only 4% of the quadrature is utilized for calculating the *J*-dependent contributions. Interestingly, for both DNA_1_ and vancomycin, this corresponds to 15k grid points for the *I*-dependent and 7k grid points for the *J*-dependent contributions within a domain. Hence, it is assumed that these favorable numbers will remain unchanged for even larger molecules.

**Figure 6:**
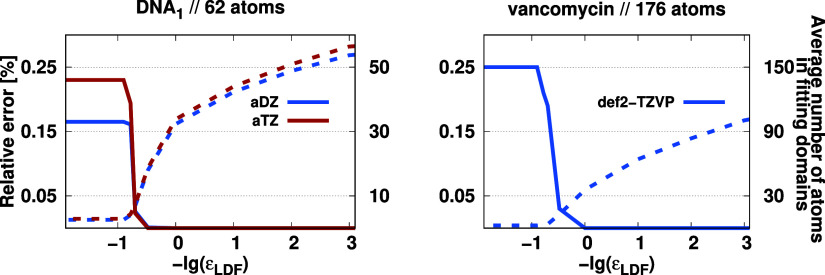
Relative error of the CABS correction (solid) and the average number of atoms in fitting domains (dashed) as a function of the corresponding truncation parameter.

Finally, the CABS correction is inspected utilizing the LDF approximation, and the corresponding ε_LDF_ parameter is determined. For these calculations, the same molecules and basis sets were used. The results are presented in Figure 6. For the DNA_1_ molecule, we see similar patterns in the relative error and the average number of atoms in the fitting domains when the aDZ and aTZ basis sets are employed. Specifically, the CABS correction is −0.19589 and −0.03191 E_h_ with the aDZ and aTZ basis sets, respectively. The approximation remains completely error-free up to a threshold value of ε_LDF_ = 3.0 au in both cases, while the relative errors are only around 0.02% with ε_LDF_ = 5.0 au. At this threshold value, the local domain contains on average only 7 atoms, which significantly reduces the computational requirements. As the threshold increases, the error rises, but even at ε_LDF_ = 10.0 au, the relative error remains under 0.25%. In this case, only the atoms selected based on Löwdin charges remain in the local fitting domain, which are formed on average of 2.5 atoms.

For the vancomycin molecule using the def2-TZVP basis set, the CABS correction is −0.134878 E_h_. Regarding the error measures, similar observations can be made; however, the error starts to increase slightly earlier compared to the previous case, which may be due to the lack of diffuse functions. In this case, the approximation is error-free up to ε_LDF_ = 1.0 au, and the relative error lies below 0.20% at ε_LDF_ = 5.0 au, which is still highly acceptable. At this threshold value, the local domains contain on average only 4 atoms, which explains the larger error compared to what we observed for the DNA_1_ molecule. Using the atom lists compiled based on Löwdin charges, the error does not exceed 0.25% in this case either. Taking into account these results, we chose a default value of ε_LDF_ = 5.0 au for the truncation parameter. This value strikes a balance between computational efficiency and the accuracy of the CABS correction, keeping relative errors within acceptable limits while optimizing the number of atoms in the fitting domains.

To provide insight into the computational requirements of the DBBSC-LNO-CCSD(T) method for systems of size similar to these molecules, the wall-clock times required for the rate-determining steps are presented using the default truncation thresholds. The results are summarized in Table 4. As can be seen, neither the DBBSC nor the CABS correction is the rate-determining step. The time required for the CABS calculations is comparable to that needed for the local MP2 (LMP2) steps, while the DBBSC calculations take significantly less time. Compared to LNO-CCSD(T), the determination of the DBBSC and CABS corrections increases the computation time required for the post-HF steps by approximately 20%. For example, for the DNA_1_ molecule with the aTZ basis set, the wall time increases from 162 to 191 min. Considering the efficiency of the method, which will be discussed in detail in the following subsection for extended systems, this overhead is highly acceptable.

**Table 4:** Wall-Clock Times (in min) Required for the Corresponding Post-HF Steps with the Default Thresholds Using Various Basis Sets

step	DNA_1_		vancomycin
	aDZ	aTZ	def2-TZVP
LMP2 correlation energy	6.2	26.4	87.0
LNO-CCSD(T) correlation energy	53.8	162.3	608.6
CABS correction	5.5	16.3	76.0
DBBSC	5.0	12.0	37.7

### 4.3 Large-Scale Applications with DBBSC-LNO-CCSD(T)

**Figure 7:**
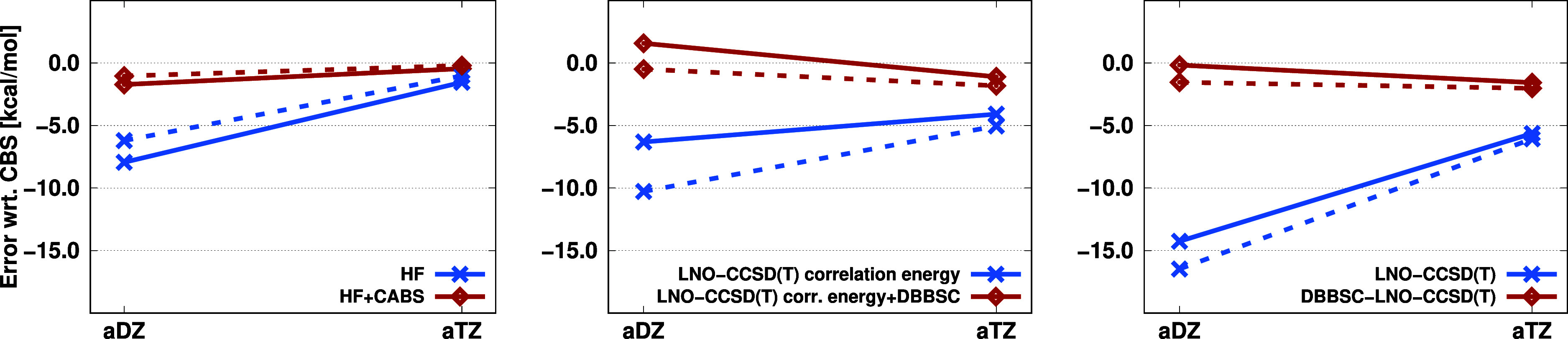
Error (in kcal/mol) of the barrier heights for the halocyclization (solid) and Michael addition (dashed) reactions using various basis sets.

In what follows, the performance of the DBBSC-LNO-CCSD(T) method is demonstrated for real-life examples where reliable CBS references are still available. First, the barrier heights are discussed, and the results are depicted in Figure 7. For the halocyclization reaction using the aDZ basis set, the CABS correction reduces the basis set error of HF from −7.94 to −1.73 kcal/mol, while the DBBSC decreases the correlation energy error from −6.32 to 1.57 kcal/mol. The error in the barrier height calculated from the total energies is −14.26 kcal/mol without corrections and −0.17 kcal/mol with corrections. As can be seen, there is a slight error compensation between the HF and correlation energies in this case, but the performance of the DBBSC-LNO-CCSD(T) method is still remarkable. The importance of the corrections is also evident with the aTZ basis set. Here, the corrections reduce the HF energy error by 1 kcal/mol and the correlation energy error by 3 kcal/mol. Overall, the LNO-CCSD(T) level basis set error with respect to the CBS reference is −5.64 kcal/mol, whereas the DBBSC-LNO-CCSD(T) method has an error of −1.57 kcal/mol. Expressing the improvement in terms of relative errors, it decreased from around 150% to 5% and from 60% to 20% with the aDZ and aTZ basis sets, respectively. A slight drawback is that the error for the DBBSC-LNO-CCSD(T) method does not decrease in this case with increasing basis set size, but this would be hard to expect after achieving almost perfect results with the aDZ basis set.

Similar results can be observed for the Michael addition reaction. In this case, compared to the previous example, the HF energy error is somewhat smaller, while the correlation energy error is more significant. Using the aDZ basis set, the HF energy error with the correction decreases from −6.21 to −1.05 kcal/mol, while the correlation energy error drops from −10.27 to −0.49 kcal/mol. Accordingly, the error in the barrier height calculated from the total energies decreases from −16.47 to −1.55 kcal/mol using the DBBSC-LNO-CCSD(T) method. With the aTZ basis set, the LNO-CCSD(T) error amounts to −6.07 kcal/mol, while with corrections, it is −2.02 kcal/mol. Inspecting the relative errors, with the aDZ basis set, it decreased by an order of magnitude, from 300% to 30%, while the improvement, 130% to 40%, with the aTZ basis set is still considerable.

**Figure 8:**
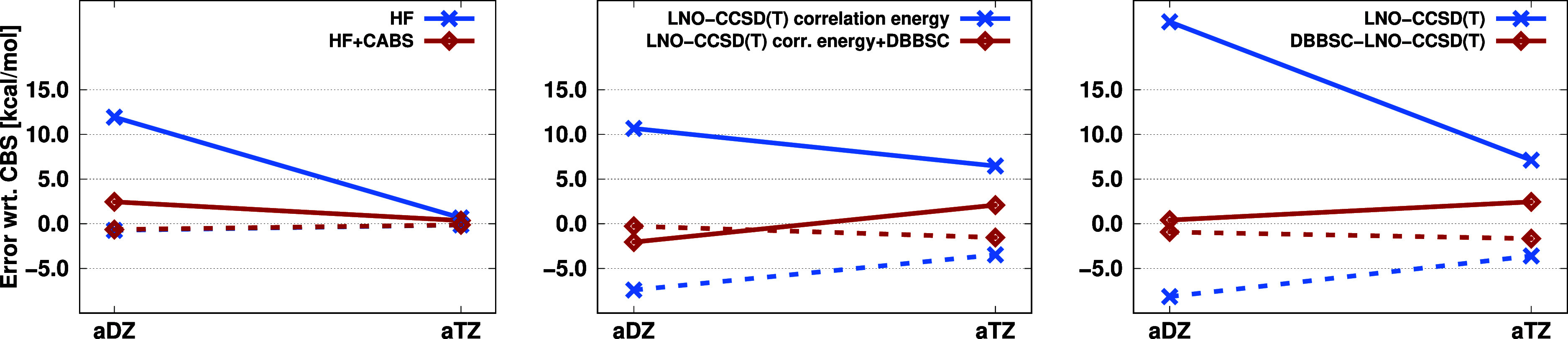
Error (in kcal/mol) of the ISOL4 isomerization reaction (solid) and the AuAmin organometallic reaction (dashed) using various basis sets.

Reaction energies were also calculated, the performance of the DBBSC-LNO-CCSD(T) method for the ISOL4 isomerization reaction and for the AuAmin organometallic reaction is summarized in Figure 8. First, the ISOL4 reaction is discussed. Starting with the aDZ basis set, the HF energy error is significantly reduced by the CABS correction, dropping from 11.93 to 2.46 kcal/mol. Without the DBBSC correction, the correlation energy error is around 11 kcal/mol, while the inclusion of DBBSC reduces it to −2.05 kcal/mol. The error in the isomerization energy obtained from the total energy is 22.60 and 0.41 kcal/mol for the LNO-CCSD(T) and DBBSC-LNO-CCSD(T) approaches, respectively. Again, a small error cancellation shows up between the HF and correlation energies for DBBSC-LNO-CCSD(T), but the magnitude of this effect is highly tolerable. For the aTZ basis set, the HF energy shows a minimal error, both with and without the CABS correction. The LNO-CCSD(T) correlation energy error is around 6 kcal/mol without the DBBSC correction, whereas it is moderated to 2.09 kcal/mol with the correction. Since the HF energy is practically error-free with these basis sets, the total energy errors match the correlation energy errors. The relative errors in this case are already smaller. The error of the LNO-CCSD(T) method is approximately 30% and 10% using the aDZ and aTZ basis sets, respectively. For DBBSC-LNO-CCSD(T), the result is practically error-free with the aDZ basis set, while the relative error is 3% with the aTZ basis set.

The difficulty of the AuAmin reaction is clearly visible. The HF energy is already close to the CBS limit using the aDZ basis set. The error is only 0.71 kcal/mol, which is reduced by 0.09 kcal/mol with the CABS correction. The correlation energy error is more significant, being 7.41 kcal/mol, which the DBBSC reduces to below 0.30 kcal/mol. Using the larger basis sets, the errors further decrease, the HF energy error is practically the same with and without the CABS correction, while the correlation energy error is halved from 3.47 kcal/mol with the correction. The error calculated from the total energies is 8.15 and 3.60 kcal/mol for the LNO-CCSD(T) method using the aDZ and aTZ basis sets, respectively, while these values are 0.92 and 1.66 kcal/mol for the DBBSC-LNO-CCSD(T) approach.

**Figure 9:**
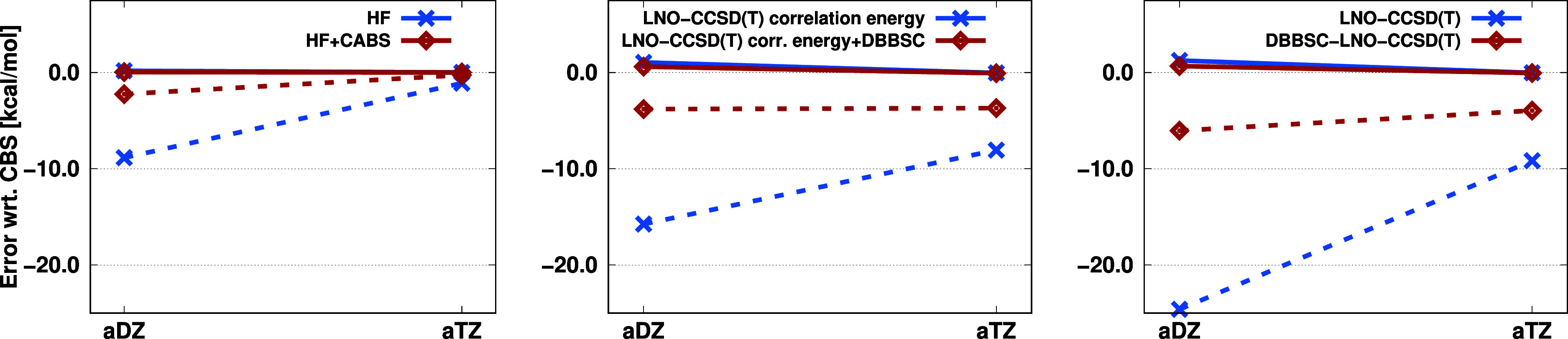
Error (in kcal/mol) of the coronene dimer interaction energy for the CP-corrected (solid) and CP-uncorrected (dashed) results using various basis sets.

The performance of DBBSC-LNO-CCSD(T) for interaction energies was also scrutinized. The results for the coronene dimer are shown in Figure 9. Here, both CP-corrected and -uncorrected results are assessed. As can be seen, the errors calculated from the CP-corrected values are nearly perfect for all methods, basis sets, and energy contributions. With the aDZ basis set, the HF energy error is 0.19 and 0.05 kcal/mol without and with the CABS correction, respectively. The correlation energy error slightly exceeds 1 kcal/mol with the LNO-CCSD(T) method, while the DBBSC correction reduces the error by half. When using the aTZ basis set, the already low errors decrease further, not exceeding 0.1 kcal/mol in any case. For the CP-uncorrected results, the errors are more pronounced. In this case, the advantage of the DBBSC-LNO-CCSD(T) method becomes evident. The HF energy error is nearly −9 kcal/mol with the aDZ basis set, and the CABS correction decreases this to −2.24 kcal/mol. An even greater improvement, –15.75 to −3.80 kcal/mol, is observed in the correlation energy. For the interaction energies calculated from the total energies, the −24.60 kcal/mol error is reduced to its quarter by the DBBSC-LNO-CCSD(T) method. When applying the aTZ basis set, the errors are smaller, but significant improvements can still be observed with the corrections. The HF energy error decreases from −1.10 to −0.26 kcal/mol, while for the correlation energy, it decreases from −8.06 to −3.69 kcal/mol. Concerning the total energy, the error is nearly −10 kcal/mol for LNO-CCSD(T), which drops to below −4 kcal/mol with the corrections. Regarding the relative errors, with the aDZ basis set, the error decreases from 100% to 25%, while with aTZ, it reduced from 40% to 15%. The benefit of evaluating DBBSC results both with and without CP-correction is that they become much closer (than without DBBSC) and their difference can be used as a tighter estimate of the remaining basis-set incompleteness.

The wall-clock times are also discussed for these representative examples. The times required for the post-HF steps are summarized in Table 5. Inspecting the results, we can conclude that the calculation of the DBBSC and CABS corrections still does not pose significant obstacles, although it varies from system to system which correction is more costly. For instance, for the halocyclization reaction using a double-ζ basis set, the determination of DBBSC is somewhat more time-consuming, while with the triple-ζ basis set, the CABS correction becomes more costly. Nevertheless, it can be seen that the overhead in both cases is around 15%. Similar conclusions can be drawn for the other examples, although the extent of the overhead varies. This is, of course, consistent with the electronic structure of the molecules and the dimensions of the local domains. The smallest overhead was obtained for the coronene dimer, where the domains are large due to the delocalized electronic structure, making the CC part relatively expensive. In this case, the overhead is about 6% regardless of the basis set. In contrast, for the isomerization reaction, the additional computational cost is around 30%, which is still acceptable considering the fairly inexpensive CC part and the overall performance of the corrections. Inspecting the favorable total wall-clock times, we can conclude that the calculation of molecular systems containing 100 atoms can be routinely performed with the DBBSC-LNO-CCSD(T) method.

**Table 5:** Wall-Clock Times (in min, Upper Panel) Required for the Corresponding Post-HF Steps Using Various Basis Sets^a^

step	halocyclization		Michael addition		ISOL4		AuAmin		coronene dimer	
	a(D+d)Z	a(T+d)Z	aDZ	aTZ	aDZ	aTZ	a(D+d)Z	a(T+d)Z	aDZ	aTZ
*E*_LNO-CCSD(T),c_	78.2	221.8	323.1	968.8	24.5	76.7	372.0	1162.5	1132.2	3113.8
*E*_CABS_	5.6	16.5	13.7	40.7	5.8	18.0	28.5	70.5	12.5	34.5
*E*_DBBSC_	7.2	15.5	25.3	66.8	2.7	5.9	43.2	117.0	57.4	160.0
										
overhead (%)	16.4	14.4	12.1	11.1	34.7	31.1	19.3	16.1	6.2	6.2

a For each example, the largest species is shown.

Illustrating the current capabilities of DBBSC-LNO-CCSD(T), interaction energies for an LTP system have been calculated. The results are collected in Table 6. Similar to the coronene dimer, the CP-corrected results are closer to the CBS references in all cases. Here, the HF energy error is only 0.6 kcal/mol, the LNO-CCSD(T) correlation energy error is 3.2 kcal/mol, and the interaction energy error calculated from the total energies is 2.6 kcal/mol due to slight error compensation. Since the CBS reference for the LNO-CCSD(T) total energy is −12.48 kcal/mol, this represents a 20% deviation when expressed as a relative error. The DBBSC and CABS corrections further improve the results. In this case, the HF error decreases to 0.5 kcal/mol, while the correlation energy error drops to 0.2 kcal/mol. As a result, the error in the total interaction energy falls below 1 kcal/mol, precisely to 0.7 kcal/mol. For the CP-uncorrected results, the differences are more significant. The CABS correction reduces the HF energy error from 21.7 to 5.7 kcal/mol, while the error in the correlation energy decreases from 59.4 to 25.9 kcal/mol with DBBSC. For the total energy, the absolute error is 81.1 kcal/mol without corrections, which reduces to 31.6 kcal/mol. This represents a 60% reduction in the errors.

**Table 6:** CABS and DBBSC Corrections to LNO-CCSD(T) Energies As Well As Basis-Set Errors of HF Energies, LNO-CCSD(T) Correlation Energies, LNO-CCSD(T) Total Energies, and DBBSC-LNO-CCSD(T) Total Energies (in kcal/mol, Upper Panel); Wall-Clock Times (in hours), and Memory Requirements (in GB) for the LTP System Using the def2-SVPD Basis Set^a^^,^^b^

	corrections		errors			
	CABS	DBBSC	HF	LNO-CCSD(T) corr. energy	LNO-CCSD(T)	DBBSC-LNO-CCSD(T)
CP-corrected	–0.11	3.34	0.59	–3.15	–2.56	0.66
CP-uncorrected	15.99	33.56	–21.68	–59.44	–81.12	–31.57
						
wall-clock time	66.9	5.5	272.5	65.9	338.4	410.8
memory requirement	196.9	5.8	14.3	20.8	20.8	196.9

a The corresponding CBS interaction energies calculated from the HF energies, LNO-CCSD(T) correlation energies, and LNO-CCSD(T) total energies are 87.94, −100.42, and −12.48 kcal/mol, respectively.

b The latter two quantities are given for the calculation of the dimer containing 1023 atoms.

To discuss the resource demands of the method, the wall-clock times and memory requirements are assessed. The calculation of the DBBSC still needs less than a 10% overhead, but the time required for the CABS correction significantly increases for such extended systems. This also assumes very compact local domains. In this case, the time required for the CABS correction is approximately equal to the time spent on computing the CC correlation energy, which is around 65 h. The total wall-clock time for the post-HF steps is approximately 6 days, while the HF calculation utilizing the LDF approximation takes more than 10 days. Therefore, solving the HF equations remains the rate-determining step, at least for such large systems and with the default LNO-CCSD(T) thresholds. Regarding memory requirements, we can conclude that the CABS correction is the bottleneck. The calculation of the correlation energy within the LNO-CCSD(T) framework requires only 21 GB of main memory, while the DBBSC correction needs an additional 6 GB. In contrast, for the construction of the Fock matrix including the CABS basis functions, where the number of basis functions exceeds 60k, the minimum required memory is around 200 GB. Nonetheless, this amount of memory is nowadays easily available, demonstrating that the DBBSC-LNO-CCSD(T) method is wide accessible, e.g., for biomolecules exceeding 1000 atoms.

## 5 Conclusions

In this study, the calculation of DBBSC was extended to large molecules. To this end, we utilized the infrastructure developed for our highly efficient LNO-CCSD(T) approach based on local approximations. In this case, the calculation of the required quantities is decomposed into the sum of contributions from individual LMOs. For these orbitals, a constrained local domain is formed, and the corresponding contributions are evaluated only within this compact subspace, thus reducing the number of variables used in the calculations. The determination of the range-separation function necessary for DBBSC, which is the rate-determining step of the procedure, is also possible by utilizing local approximations. This only required relatively minor modifications to the existing infrastructure established for LNO-CCSD(T) calculations, which should be also possible for other local correlation approaches. Furthermore, the LDF approximation was applied to the calculation of the CABS correction. In this case, the local fitting domain contains only the atoms and their associated auxiliary functions necessary for the accurate fitting of the integrals of the given LMO thereby significantly speeding up the calculations.

The numerical quadrature required for the DBBSC calculation was carefully examined. The computation time needed to determine the range-separation parameter linearly depends on the number of grid points. Since the grid can be very large in general applications, it is advisable to select the smallest possible mesh to minimize the costs. We demonstrated that using TA*n* quadratures for the correction, the smallest grid is highly sufficient. The largest difference between the TA1 and TA5 quadratures is only 0.02 kcal/mol in thermochemical properties determined for the KAW benchmark set. This finding was also confirmed for larger molecules. Subsequently, we demonstrated that the local approximations used for LNO-CCSD(T) calculations are adequately accurate for determining the range-separation function and the DBBSC correction. For extended systems, it was proven that the error from the local approximation is around 3–4 μE_h_/atom employing the default (Normal) parameter settings. In addition, efficient prescreening techniques were presented for grid compression. With these practically error-free procedures, the grid points used within the domain can be significantly reduced. Applying the proposed preI+preJ scheme, on average, 15k grid points are needed for the less costly *I*-dependent contributions, while 7k grid points are required for the rate-determining *J*-dependent contributions within the domains. The error of this procedure for the DNA_1_ and vancomycin molecules ranges from 30 to 50 μE_h_, which, when expressed as a relative error, is negligibly small. Then, the error of the LDF approximation for the CABS correction was determined. It was shown that for basis sets containing diffuse functions, the local fitting domain contains on average 7 atoms, with an expected relative error of approximately 0.02%. For basis sets without diffuse functions, the domains contain fewer atoms, thus the error begins to increase earlier, but even with the minimal atom list selected solely based on Löwdin charges, it did not exceed 0.25%.

Using the defined truncation parameters, the efficiency of the DBBSC-LNO-CCSD(T) method was demonstrated for extended systems of 100–1000 atoms. For this purpose, barrier heights, reaction energies, and interaction energies were calculated for real-life examples where reliable LNO-CCSD(T)/CBS references were available. Based on the numerical results, we concluded that the corrections drastically reduce the basis set incompleteness error, especially when double-ζ basis sets are used. In these cases, the error often did not exceed 1 kcal/mol, but significant improvements can also be achieved with triple-ζ basis sets. A minor drawback is that the error of the DBBSC-LNO-CCSD(T) method does not always decrease monotonically with increasing basis set size, but this is hard to expect after the almost perfect double-ζ results. Nevertheless, the corrections always improve the results, regardless of the quality of the basis set used. Considering the computational overhead in practical applications, the calculation of the post-HF steps takes only 5–30% more time compared to LNO-CCSD(T) calculations, which allows the accurate description of extended molecular systems within a reasonable computational time frame.

## Supporting Information Available

Calculated total, atomization, reaction, and interaction energies (ZIP)
